# Effects of extrusion process conditions on nutritional, anti‐nutritional, physical, functional, and sensory properties of extruded snack: A review

**DOI:** 10.1002/fsn3.4472

**Published:** 2024-10-18

**Authors:** Ibrahim Mohammed Ali, Sirawdink Fikreyesus Forsido, Chala G. Kuyu, Endris Hussen Ahmed, Kumsa Negasa Andersa, Kasech Tibebu Chane, Tolina Kebede Regasa

**Affiliations:** ^1^ Department of Plant Science, College of Dry Land Agriculture Samara University Samara Ethiopia; ^2^ Department of Postharvest Management, College of Agriculture and Veterinary Medicine Jimma University Jimma Ethiopia; ^3^ Department of Agro Processing Holeta Polytechnic College, Holeta College Holeta Ethiopia

**Keywords:** Extrusion cooking, Nutrient‐rich, Optimization, Processing condition, Sensory properties, Snack food

## Abstract

Consumers' demand for ready‐to‐eat extruded snack is increasing due to their convenience and time saving. Extrusion cooking is the most commonly used technology for manufacturing ready‐to‐eat snack products. However, improper management of extrusion parameters such as the barrel temperature, feed moisture content, pressure, and other parameters of the extruder can influence the quality of extruded snack foods. This paper aimed to review the effects of extrusion process conditions on the quality of the extruded snack. According to this review, most of the nutritional composition and anti‐nutritional factors are sensitive to the extruder's high barrel temperature. High barrel temperature decreased the anti‐nutritional factors of the extrudates. Low feed moisture content and high barrel temperature minimize the bulk density and hardness while increasing the extruded snack's expansion ratio and water solubility index. In contrast, low barrel temperature increased the color and appearance of the extrudates while decreasing the flavor and overall acceptability of the extrudates. In general, this review indicated that extrusion processing parameters need to be optimized to deliver nutrient‐rich extruded snack with appropriate physical, functional, and sensory properties.

## Graphical abstract

Extrusion process variables have a major impact on nutritional, anti‐nutritional, physical, functional, and sensory properties of extruded snack.

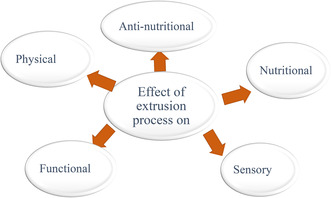

## 1 INTRODUCTION

Snacks are common names for small portions of food eaten in between meals. The definition of a snack might vary depending on the time of day, the type or quantity of food consumed, or even the location (Hess et al., 2016). In general, snacks include different things eaten or consumed in between meals and include sandwiches, fresh fruit, sweets, and crisps. Snack foods currently make up a sizable portion of the global population's diet and are among the most popular extruded foods in terms of commerce. Owing to its affordability and ready‐to‐eat nature, it has seen an increase in consumption over the past 25 years among all age groups, but especially among youngsters (Sebastian et al., 2008). Due to gelatinization properties, extrusion cooking is mostly employed to produce snacks, which are often manufactured from cereal flour or starches (Wandee et al., 2014). Thailand is one of the top exporters of food products worldwide.

Extrusion is a form of thermal cooking in which mixtures of raw materials are forced through a screw die under various pressures, temperatures, and mechanical shear to produce the desired output (Alam et al., 2016). Extrusion technology has been used to generate several food products, including pasta, breakfast cereals, bread crumbs, biscuits, crackers, croutons, chewing gum, baby foods, snack foods, confectionery items, dried soups, and dry beverage mixes. Extrusion can break covalent and non‐covalent interactions between ingredients in complex food matrices, retain nutrients, and change functional properties. Additional benefits include decreased lipid oxidation, increased soluble dietary fiber, and decreased ANFs (Singh et al., 2007).

**FIGURE 1:**
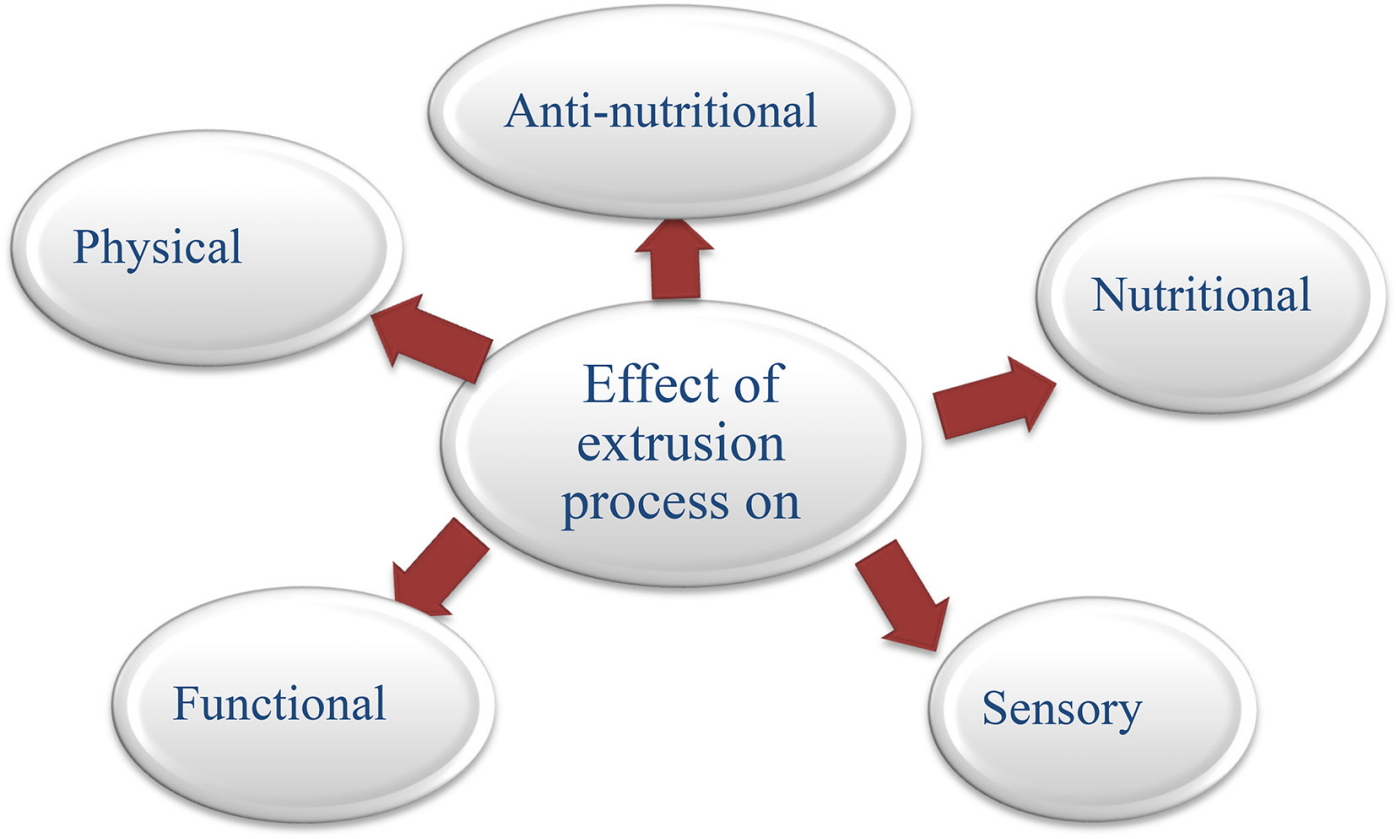
Graphical abstract of the review paper.

It is vital to remember that the processing parameters used during extrusion cooking can significantly affect the end product's nutritional content and other attributes. For instance, if the temperature in the barrel is too high, the products may lose nutrients and darken, and if it is too low, the products may not expand, which is undesirable for snack foods. Alternatively, if the feed moisture content is excessively high, an inferior expanded product is produced. The goal of this paper was to examine the effects of the extrusion processing parameters on the overall quality characteristics of the extruded snack (Figure 1).

## 2 EFFECTS OF EXTRUSION PROCESS CONDITIONS ON NUTRITIONAL, ANTI‐NUTRITIONAL, PHYSICAL, FUNCTIONAL, AND SENSORY PROPERTIES OF EXTRUDED SNACK

### 2.1 Extrusion cooking machine technology

The food business has access to various food production and processing technologies to create numerous food products. Extrusion processing is one of the most popular processing techniques used today. Extrusion is a high‐temperature, rapid process that combines a number of unit actions (such as mixing, cooking, forming/kneading, and shearing) to transform raw ingredients into a range of food products (Bordoloi & Ganguly, 2014).

A feeding device (hopper), which can feed directly into the extruder intake or a preconditioned area where temperature and moisture adjustment take place, is used to feed raw materials (such as cereal or pulse flour) into the extruder. Using a hopper, the dry mixture of raw materials was first delivered into the extruder barrel, properly combined with other ingredients or food additives, and then given proper preconditioning using water or steam and occasionally low‐temperature heating (Nikmaram et al., 2017).

**FIGURE 2:**
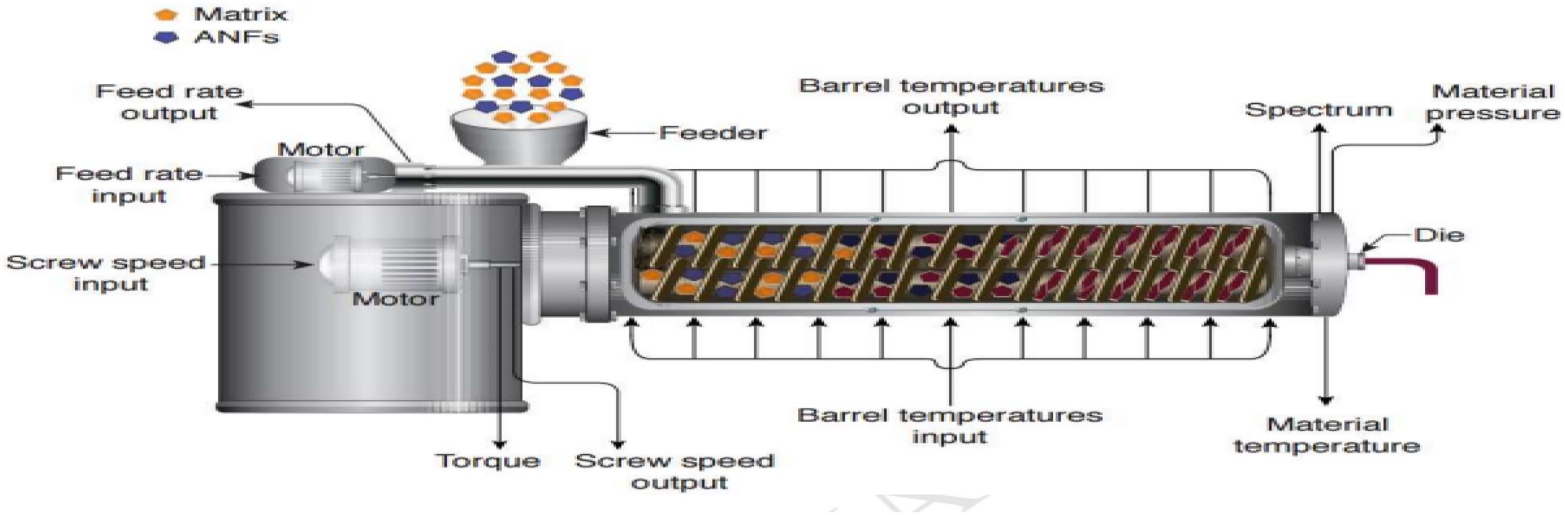
A graphical example of a typical food extruder system (Patil et al., 2016).

**FIGURE 3:**
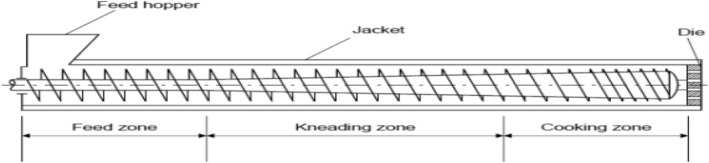
Illustration of a single screw extruder (Riaz, 2013).

The screw along the barrel length contributes by moving, compressing, and kneading the extrudates into plasticized matter. Here, extrudates undergo high‐temperature short processing cooking (>100°C) under increasing pressure and shearing force from the barrel screw. Cooked extrudates flow through a small die at the end of the barrel that has been designed or shaped to produce the final extruded product. The finished product is then cut with a sharp blade to the desired size or length (Kaur et al., 2014). The most commonly used extruders in the food industry are twin‐screw (Figure 2) and single‐screw extruders (Figure 3; Mościcki, 2011).

### 2.2 Effects of extrusion processing parameters on anti‐nutritional factors

Extrusion conditions, such as screw speed, barrel temperature, feed moisture content, and extrusion pressure, all have a significant impact on how well anti‐nutritional factors are reduced. The screw of an extruder affects anti‐nutritional factors levels as well as the degree of heating, starch gelatinization and dextrinization, and protein denaturation. Tadesse et al. (2019) documented a reduction in anti‐nutritional factors such as phytate and tannin of the extruded snack as the barrel temperature rose in their sorghum‐based extruded product. The author stated that the phytate content of the extrudates reduced from 26% to 50% as the barrel temperature in the extruder upgraded from 135°C to 165°C.

It has been demonstrated that an extrusion temperature of 140°C is effective in inducing a noticeable drop in this anti‐nutritional factor in wheat and rice bran concerning the decrease in heat‐stable anti‐nutritional factors such as trypsin inhibitors (Kaur et al., 2015). Likewise, compared with the finished product extruded at a temperature lower than 140°C, Jatropha *curcas* was able to eliminate both lectin and trypsin inhibitors (Valdez‐Flores et al., 2016).

When the feed moisture content is increased (14 to 22%) during extrusion, significant variations in anti‐nutritional factor levels may occur. The reduction of phytate, tannin, and polyphenol in lentils has been found to be more pronounced in feed materials with increased moisture content compared with feeds with lower moisture content (Rathod & Annapure, 2016). Furthermore, Kaur et al. (2015) demonstrated that the best processing conditions for lowering the phytate level of wheat bran involve high feed moisture content (20%) combined with a moderate extrusion cooking temperature (115°C).

### 2.3 Effects of extrusion processing parameters on nutritional values of extrudates

#### 2.3.1 Starches

In the case of starch, high temperature and pressure can cause the starch granules to gelatinize and form a melt. The process of extrusion can affect the pace and degree of starch digestion from a nutritional standpoint. This is because the structure of the starch molecules can be modified during extrusion, which can affect how easily they are broken down by enzymes in the digestive system. For example, extruded foods may have a higher degree of starch gelatinization, which can lead to more rapid starch digestion and absorption in the small intestine (Singh et al., 2007). According to Péronnet et al. (2015), extruded products often have excellent starch digestibility. The features of starch digestion may be impacted by starch fragmentation during extrusion, particularly waxy starches. Dextrinized starch has a higher propensity for retrogradation because of its increased molecular mobility (Zhang et al., 2014). Wang et al. (2015) have reported that retrogradation during extrusion cooking can result in the formation of enzyme‐resistant starch (RS) in cereals or cereal starches.

#### 2.3.2 Dietary fiber

The non‐digestible components of plant cell walls, including lignin, waxes, cutin, and suberin, as well as polysaccharides (cellulose, hemicellulose, mucilage, oligosaccharides, and pectin), have historically been referred to as dietary fiber. Extrusion increases the solubility and availability of dietary fiber by breaking down the cell walls and other structural components of the material. In the case of waxy barley flour, extrusion has been shown to increase the amount of soluble dietary fiber, which contributes to an overall increase in the total dietary fiber content. A research conducted by Sobota et al. (2010) indicated that extrusion process tends to enhance the soluble fraction of dietary fiber (SDF) content. In a different study, soluble dietary fiber (SDF) was improved from oat bran by using extrusion. SDF from extruded oat bran was found to have more aggregates, a higher gelatinization temperature, higher solubility, swelling capacity, and solvent retention capacity, as well as a higher apparent viscosity and consistency coefficient, a lower flow behavior index, and improved foam ability when compared with SDF from untreated oat bran. The functional qualities of SDF made from oat bran are enhanced using the extrusion process (Zhang et al., 2011).

#### 2.3.3 Protein quality and solubility

Changes in essential amino acid availability and digestibility have had a significant impact on proteins. A recent study showed that the extrusion process has improved the digestibility and availability of critical amino acids in food products. Extrusion may be an effective way to improve the nutritional quality of lentils and other legumes by increasing the availability and digestibility of essential amino acids (Nosworthy et al., 2018).

In addition, extruded maize flour and soy protein concentrates significantly increased the biological value of proteins while maintaining the same levels of important amino acids, including accessible lysine (Omosebi et al., 2018). Inactivation of anti‐nutritional chemicals and the thermal denaturation of proteins have been identified as the main causes of improvement in digestibility after extrusion.

Protease inhibitors, which attach to enzymes such as trypsin and prevent them from digesting proteins, can also be found in legumes and cereals. The action of these interfering chemicals can be greatly reduced and protein digestion can be promoted by high extrusion temperature combined with shear, according to numerous studies (Kaur et al., 2015). Because proteins make dough more viscous and inhibit flow in the extruder barrel, high‐protein raw materials expand less during extrusion and produce less expanded products (Pitts et al., 2014). Accordingly, protein functionality during extrusion has not focused on expansion but rather on solubility and texturization. Despite the advantage, the presence of Millard reaction that minimizes the nutritional value of the protein is a drawback of extrusion cooking. As a complex multivariate process, the extrusion parameter should be controlled if product quality needs to be maintained (Singh et al., 2007).

#### 2.3.4 Lipids

During the extrusion process, proteins and starch can form complexes with lipids, creating a variety of structures that can affect the nutritional and functional properties of the final product. In particular, it binds to starch and becomes significantly more difficult to remove with nonpolar solvents. The formation of these complexes can have both positive and negative effects on the nutritional and functional properties of the final product. The complexation of proteins and starch can improve the digestibility and bioavailability of essential amino acids and other nutrients. However, it can also lead to decreased solubility and increased viscosity, which may affect the texture and sensory characteristics of the product (De Pilli et al., 2012). The authors also discovered that high feed moisture content and barrel temperature promoted the development of starch‐lipid complexes. The oxidative stability of the lipids is enhanced by their binding to starch. As a result, compared with lipids that are not bonded, a significant fraction of critical fatty acids will be maintained during extrudate storage.

### 2.4 Effects of extrusion processing parameters on functional properties of the extrudates

#### 2.4.1 Water absorption index

The water absorption index indicates the availability of molecules of hydrophilic groups (such as hydroxyl) to interact with water molecules. Water Absorption Index (WAI) in g/g is a measure of the ability of the samples to absorb water, by indicating the water available for gelatinization and other physicochemical changes (Osibanjo et al., 2017). The influence of extrusion conditions on the water absorption index has been reported by many authors. Previous studies have documented how the water absorption index increases as the moisture content increases. The increased percentage of gelatinized starch granules was blamed for the rise in the water absorption index, and the highest water absorption index value showed total gelatinization (Srivastava & Genitha, 2012). It has also been documented that the water absorption index increases when the extrusion temperature enhances (Singh et al., 2016).

#### 2.4.2 Water solubility index

The water solubility index measures the number of soluble polysaccharides that are released from starch granules and dissolved in water (Maseta et al., 2017). The degree of dextrinization or degradation of the material can be determined by measuring the WSI, which may provide important information regarding its appropriateness for different uses in the food sector.

Rising temperature during extrusion cooking causes molecular breakdown, which raises the water solubility index (Srivastava & Genitha, 2012). In other words, the water solubility index monitors the starch degradation caused by extrusion cooking and can be used as a biomarker of the breakdown of molecular components. The water solubility index has also been reported to increase with an increase in extrusion temperature and a decrease in moisture content. The high level of starch breakdown that leads to a greater release of soluble chemicals may be the cause of water solubility index to rise with extrusion temperature (Singh et al., 2016). The decrease in the water solubility index with an increase in moisture content may be attributed to lateral expansion because of plasticization of the melt and less degradation of starch.

### 2.5 Effects of extrusion processing parameters on physical properties of extrudates

#### 2.5.1 Expansion ratio

The expansion ratio indicates the degree of puffing sample experiences after it leaves the extruder. It has been discovered that the main element influencing the expansion ratio is the feed moisture content. When feed moisture was increased from 10% to 13%, the expansion ratio increased. When feed moisture was increased to 14%, the expansion ratio dropped (Srivastava & Genitha, 2012). The radial expansion of the extruded products is influenced by various factors, including barrel temperature and feed moisture content. When the barrel temperature is increased, the water inside the feed material undergoes greater superheating, which leads to increased steam formation and consequently more expansion. On the other hand, reducing the feed moisture content can also lead to increased radial expansion because the available water will be superheated to a higher degree during processing (Gopirajah & Muthukumarappan, 2018).

#### 2.5.2 Hardness

Hardness refers to the breaking force of the extrudates required to fracture. The extrusion process parameters have a significant influence on the hardness of the extrudates. Wondimu and Admassu Emire (2016) mentioned increasing barrel temperature and decreasing feed moisture content result decreased in hardness. This is because greater temperatures cause more expansion, which causes extrudates to puff up and become less dense, softening the final product. And also increasing the feed moisture content tends to increase the breaking strength of the extrudates. This is due to the increase in density of the extrudates since denser products are resistant to break (Wondimu & Admassu Emire, 2016).

#### 2.5.3 Bulk density

Bulk density is a significant parameter because it influences the packaging material. It dictates the characteristics of the container or package, and product density influences the amount and strength of packaging material, texture, and mouth feel (Agrawal et al., 2013). The barrel temperature and feed moisture content of extruder affect the bulk density of the extrudate. Mohammed et al. (2024) reported an increase in bulk density of the extrudates as the barrel temperature reduced and feed moisture content enhanced in their extruded snack developed from finger millet, sweet potato, and soybean composite flours. Numerous studies have noted that high barrel temperature and low feed moisture extrusion conditions typically result in substantially expanded extrudates with decreased bulk density and hardness (Meng et al., 2010). In general, a decrease in bulk density would follow a rise in temperature because extrusion heating causes more expansion and gelatinization.

### 2.6 Effects of extrusion processing parameters on sensory properties of extrudates

The sensory properties of extrudates, or the characteristics perceived by our senses (taste, texture, aroma, appearance), can be significantly influenced by the extrusion conditions. Significant reductions in color and aroma values of whole pearl millet‐based extruded snacks at higher extrusion barrel temperatures were documented by Sobowale et al. (2021). This might be due to the mild browning reaction, gelatinization/breakdown of starch molecules, or relative interactions within these modifications at elevated temperatures and pressures in conjunction with the in‐barrel shearing effect during extrusion. The extrusion conditions can affect the color and visual appearance of the extrudates. Temperature plays a crucial role in browning reactions, which can lead to desirable color development in certain products.

In addition, changes in processing parameters can affect the surface smoothness, shape, and size of the extrudates, influencing their overall appearance. The flavor and aroma of extrudates can be influenced by factors such as ingredients used, processing temperature, and residence time during extrusion. High temperatures and longer residence times can lead to flavor and aroma changes, including Millard reaction products that contribute to a nutty or toasted flavor (Tadesse et al., 2019). A significant reduction in the crispness of the extrudates at lower feed moisture content was indicated by Mohammed et al. (2024). The possible reason is the increase in density and hardness of the extrudates at maximum feed moisture content. Overall the extrusion processing condition has a significant impact on the sensory quality of the extruded snack.

## 3 CONCLUSION

Extrusion is a type of thermal processing that applies several different processes, including high temperature, high pressure, and shear forces to an uncooked mass, such as cereal meals, to produce goods such as snacks, ready‐to‐eat cereals, confections, and crisp breads. The processing parameters of the extruder's machine have a significant effect on the quality of the finished products. This thermal process affects the end product components in various ways, both positive and negative. Extrusion cooking improves nutrition digestibility and starch gelatinization while also modifying the functional qualities of the finished products. In contrast, the adverse impacts include the potential loss of protein, particularly lysine, and discoloration of the finished goods. The detrimental effects of extrusion are typically experienced when processing conditions are either too high or too low. From the extrusion processing parameters, the barrel temperature and feed moisture content have a considerable impact on the characteristics of the final product than others. Therefore, to produce high‐quality extruded goods with acceptable sensory properties, careful management of the extrusion processing parameters is essential.

## AUTHOR CONTRIBUTIONS

**Ibrahim Mohammed Ali:** Conceptualization (lead); formal analysis (equal); validation (equal); writing – original draft (equal); writing – review and editing (equal). **Sirawdink Fikreyesus Forsido:** Conceptualization (equal); validation (equal); visualization (equal); writing – original draft (equal); writing – review and editing (equal). **Chala G. Kuyu:** Conceptualization (equal); validation (equal); visualization (equal); writing – original draft (equal); writing – review and editing (equal). **Endris Hussen Ahmed:** Validation (equal); visualization (equal); writing – original draft (equal); writing – review and editing (equal). **Kumsa Negasa Andersa:** Validation (equal); visualization (equal); writing – original draft (equal); writing – review and editing (equal). **Kasech Tibebu Chane:** Validation (equal); visualization (equal); writing – original draft (equal); writing – review and editing (equal). **Tolina Kebede Regasa:** Validation (equal); visualization (equal); writing – original draft (equal); writing – review and editing (equal).

## CONFLICT OF INTEREST STATEMENT

Authors declare that they have no conflict of interest.

## ETHICS STATEMENT

Ethics approval was not required for this research.

## Data Availability

Data are available upon request from the corresponding author.
